# Intelligent industry, energy regulation and ecological transformation—Taking equity financing as the moderating variable

**DOI:** 10.1371/journal.pone.0294783

**Published:** 2024-02-14

**Authors:** Yunyi Wu

**Affiliations:** Gas Company of Sinopec, Beijing, 100029, PR China; Universiti Teknologi MARA, MALAYSIA

## Abstract

With the panel data of 21 China’s industrial industries from 2008 to 2020, the relationship models between intelligent industry, energy regulation and ecological transformation are constructed and tested from two dimensions of resource saving and environmental friendliness, then equity financing is introduced into this model as moderating variable to discuss the moderating effects on the relationships between intelligent industry, energy regulation and ecological transformation. Results show that: ⑴China’s industrial industries significantly transformed to the resource-saving type, and the environment-friendly level stayed in a slow progression. ⑵Intelligent industry affected ecological transformation positively and significantly. The impact of energy regulation on ecological transformation was nonlinear. The regulation of energy consumption can significantly stimulate the transformation of resource saving, and restrain the transformation of environmental friendliness; the regulation of energy structure can significantly stimulate the transformation of environmental friendliness. ⑶ Equity financing can positively moderate the relationship between intelligent industry and ecological transformation, and it can also moderate the regulation of energy structure and promote the transformation to environmental friendliness, especially in the low consumption industries.

## 1. Introduction

In the recent years, China has made great efforts in ecological and environmental protection, emphasizing and formulating environmental regulations in China’s 13^th^ five-year plan, the report of the 19th National Congress of the Communist Party of China, and so on. At the 13th National People’s Congress held in 2019, ecological protection was once again regarded as one of the key issues. In recent years, China has witnessed gradual improvements in its ecological environment. However, the country’s environmental performance still lags behind on a global scale. According to the "2020 Environmental Performance Index (EPI)" jointly released by institutions such as Yale University in the United States, China ranks 120th out of 180 countries. Specifically, its air quality ranks 137th, and its ecological vulnerability ranks 156th, all of which are considered subpar. Furthermore, during the COP26 (Conference of the Parties to the United Nations Framework Convention on Climate Change) held in 2021, reducing coal consumption was included in the convention for the first time. As the world’s largest carbon emitter, China continues to face the daunting challenge of ecological transformation. For the industrial sector, intelligent transformation is also urgent. Since Germany proposed ‘Industry 4.0’ in 2013, China has also issued ‘Made in China 2025’. The dual transformation of ecological and intelligent industries has led to an increasing demand for financing, and the regulatory role of financing on variables related to ecological transformation has become increasingly important.

The goal of ecological transformation is to build a green development system, with the core of resource conservation and environmental friendliness [1]. Therefore, ecological transformation is a transition to resource conservation and environmental friendliness. Resource conservation is to reduce energy and material consumption, while environmental friendliness is to decrease pollution emissions, protect and repair the ecological environment, which is the main task of building a "two oriented society" in China. There are many driving factors for ecological transformation, including technological progress, investment, openness, corporate profitability, the use of green energy, environmental regulations, and so on. Technological development has promoted social development and ecological improvement, so both green technological innovation [2] and overall technological progress [3] can better drive ecological transformation. In the process of rapid economic development in China, the characteristics of investment driving are relatively obvious, and a large amount of energy consumption and pollution emissions have also been generated [4]. Therefore, fixed capital investment is also one of the factors affecting ecological transformation, economic globalization can also improve energy efficiency to a certain extent, which is conducive to ecological transformation [5,6], while the level of environmental protection investment is a direct force for environmental governance, directly determining the extent of environmental improvement. The degree of openness also has an impact on the ecological environment. Foreign direct investment drives external technology spillovers, which can promote the improvement of the ecological environment. The use of green energy can support the realization of ecological transition by reducing the stock of carbon dioxide emissions [7–9], and there are abundant relevant studies, including studies on China [10], G-7 [11], the United States [12], Canada [13] and other countries. The impact of environmental regulation on ecological transformation is relatively complex, and relevant research is relatively abundant [2,14–17]. Quantitative methods mainly use indicators such as environmental administrative penalties, the proportion of environmental investment, and the rate of pollutant treatment. Relevant research generally focuses on environmental regulation in terms of environmental friendliness, but lacks environmental regulation in terms of resource conservation. Environmental regulations on resource investment mainly focus on energy consumption, including energy-saving "dual control" and energy structure adjustment policies. Currently, there is a lack of research on the impact of energy regulations on ecological transformation. In ecological transformation, financing is an important source of investment, so the regulatory role of financing on other influencing factors cannot be ignored. More financing can promote green technology innovation, pollution control, etc., among which equity financing is more in line with the capital requirements in ecological transformation [18,19]. Equity financing can directly promote R&D investment, environmental protection investment, etc. [19,20]. However, how to adjust the relationship between intelligent industry, energy regulation, and ecological transformation needs further research.

The marginal contributions of the paper include: (1) Different from previous studies, we take equity financing into consideration and construct a comprehensive relationship model including intelligent industry, energy regulation, and ecological transformation, which can discover the moderating effect of equity financing. then, we conduct empirical tests to supplement existing research on ecological transformation. (2) The previous studies always only use single indicator such as ecological efficiency or pollutant emissions to quantify the level of reginal ecological transformation, which will lead to biased evaluation easily. In fact, the ecological transformation includes both resource conservation and environmental friendliness. Therefore, we quantify ecological transformation from two dimensions which can avoid the biased evaluation. (3) The previous studies focus on the effect of environmental regulation on reginal ecological transformation. In fact, the environmental regulation only focuses on environmental friendliness while ignoring the effect of resource conservation. We introduce energy regulation variables which can take energy regulation and environmental regulation into consideration simultaneously.

## 2. Theoretical analysis and research hypothesis

### 2.1 Intelligent industry and ecological transformation

The concept of intelligent industry originated from the German "Industry 4.0" plan, characterized by mechanization, automation, informatization, and networking. It continuously integrates various terminals with environmental awareness capabilities and various technologies into all aspects of industrial production, significantly improving production efficiency, reducing product costs and resource consumption, and upgrading traditional industry from automation to a new stage of intelligence [21]. Intelligent industry has an important impact on industrial development, which can significantly improve production efficiency, reduce production costs, and promote industrial transformation and upgrading [22]. At the same time, research shows that in the process of industrial transformation and upgrading, intelligent industry development can generate industrial efficiency improvement effects, industrial structure optimization effects, and industrial ecological environment improvement effects. Intelligent production systems can maximize resource allocation and manage energy consumption, reduce unnecessary factor input, promote energy conservation, emission reduction, and green development [23,24]. Intelligent industrial development and energy transformation can also form a good interactive effect, promoting the transformation of the industrial industry towards low-carbon and clean new energy [25]. It can be seen that technological innovation in intelligent industry can produce "ecological environment improvement effects", leading the industrial industry to transform towards a green industry. On the other hand, the development of intelligent industry can also lead to changes in the organizational structure of enterprises. Chi Renyong et al. [26] have shown that the matching of organizational structure changes and intelligent industry can greatly improve the performance of enterprises. With the improvement of organizational management efficiency and decision-making efficiency, resource utilization efficiency can be significantly improved, leading to the transformation of enterprises in resource conservation. In summary, it can be seen that the development of intelligent industry can promote the ecological transformation of enterprises from two dimensions: technological innovation and organizational change. Therefore, the following hypothesis can be proposed:

H1a: The development of intelligent industry can improve the efficiency of resource utilization, thereby promoting the transformation of the industrial industry to a resource saving type.
H1b: The development of intelligent industries can reduce undesired output, thereby promoting the transformation of industrial industries to environmentally friendly ones.

### 2.2 Energy regulation and ecological transformation

The conclusion that environmental regulation can promote a significant improvement in the ecological level has been more confirmed. The impact of the intensity of environmental regulation on the ecological transformation of enterprises may be uncertainty. Porter’s hypothesis believes that appropriate environmental regulation will stimulate the creativity of enterprises, and the compensation effect obtained through innovation can compensate for the costs incurred by enterprises in complying with environmental regulations. On the other hand, the cost paid by enterprises to comply with environmental regulations can also form a crowding out effect on innovation investment, thereby inhibiting the ecological transformation of enterprises. It can be seen that there is a "threshold" effect in the intensity of environmental regulations [16]. For example, Ju Ke et al. [17] found that the impact of environmental regulation intensity of green total factor productivity (GTFP) showed significant industry heterogeneity between 2003 and 2015. In heavily polluted industries, environmental regulation was in the stage of suppressing GTFP, while in lightly polluted industries, it was in the stage of encouraging GTFP. In terms of agricultural ecological environment, environmental regulation can drive the convergence of agricultural environmental efficiency in the short term, while the direct driving effect is significantly negative in the medium to long term. Therefore, the intensity of environmental regulation has a significant impact on the stimulating effect of agricultural environmental technology spillovers. It can be seen that environmental regulation has nonlinear characteristics.

In relevant studies, environmental regulation has focused on pollution emissions, while ignoring the regulation of resource inputs. Energy is an important source of carbon emissions, and energy regulation is also an important component of environmental regulation. Therefore, the intensity of energy regulation will also have the same stimulating effect on energy technology inputs and technology spillovers, thereby promoting or suppressing ecological transformation. Research has shown that energy conservation biased technology progress can have an important impact on energy consumption and carbon emissions, and the promotion and application of energy conservation biased technology can help promote China’s industrial industry to cross the "threshold" of energy conservation and emission reduction. However, from 1995 to 2015, it was still in the inhibition stage and has not yet crossed the "threshold" [27]. Energy is an important resource input, and energy regulation can have a direct influence on the level of resource conservation in ecological transformation, while it can also have an indirect impact on the level of environmental friendliness. Research has found that the energy consumption effect is one of the important factors of atmospheric pollution [28], and energy use efficiency is also an essential influencing factor of environmental pollution effects in the process of urbanization [29]. Energy regulation can promote enterprises to reduce energy consumption improve energy efficiency, reduce environmental pollution, and thereby enhance the level of environmental friendliness. It can be seen that energy regulation can have an important impact on ecological transformation, and it is likely to have nonlinear characteristics similar to environmental regulation. Therefore, the following hypothesis are proposed:

H2a: Energy consumption regulation can have a significant impact on the level of resource conservation and has nonlinear characteristics.
H2b: Energy consumption regulation can have a significant impact on the level of environmental friendliness and has a non-linear characteristic.
H2c: Energy structure regulation can have a significant impact on the level of environmental friendliness and has nonlinear characteristics.

### 2.3 Intelligent industry, energy regulation, equity financing, and ecological transformation

According to the resource-based perspective, industrial sectors need sufficient financial resources to carry out green technology innovation. D’Orazio and Valente [18] have found that capital plays a significant role in the development and dissemination of green technology. In an environment where the public has urgent requirements for ecological transformation, financing is more conducive to the dissemination of green technology, which shows that adequate funding is of great significance for ecological transformation. Compared to indirect financing and debt financing by commercial banks, equity financing is more in line with the inherent requirements of technological innovation. Enterprises are under no pressure to pay debts and interest, meeting the continuous and cumulative needs of technological innovation. At the same time, shareholders’ participation in management can improve the efficiency of capital utilization [19]. Equity financing also meets the inherent requirements of green technological innovation, according to the pecking order financing theory, Equity financing is more conducive to green technology innovation in ecological transformation.

Intelligent industry is the direction of the current transformation of traditional industries, which can significantly improve production efficiency and create more profits for enterprises. When enterprises have sufficient funds, they are willing to conduct continuous technological upgrading and intelligent transformation. Applying equity financing to intelligent transformation also meets the interests of shareholders. The improvement of intelligent level can achieve a win-win effect of improving production efficiency and ecological level, therefore, the following hypothesis can be proposed:

H3: Equity financing positively regulates the relationship between intelligent industry and ecological transformation, and the more funds raised through equity financing, the more significant the effect of intelligent industry on resource-saving level and environment-friendly level.

Energy regulation will stimulate enterprises to reduce consumption and make energy restructuring to avoid increasing compliance costs, thus improving environmental quality and accelerating ecological transformation. When companies are under great financial pressure, the willingness to ecological transformation will be significantly reduced, the intensity of energy regulation has a disincentive effect on ecological transformation since ecological transformation is characterized by long cycles and high costs. The low-cost and long-cycle characteristics of equity financing can meet the demand for funds for technological innovation in ecological transition and produce a better smoothing effect. When equity financing is more adequate, equity investors can tolerate the risks in energy transition and are prepared to pay the costs arising from energy regulation [30], and obtain a risk premium. Energy regulation has a continuous character, and it has become a consensus for enterprises to fulfill their social responsibility and follow energy regulation. When internal financing is insufficient, equity financing can provide a guarantee for enterprises to comply with energy regulation, thus equity financing can positively regulate the relationship between energy regulation and ecological transition. This leads to the hypothesis that:

H4a: Equity financing positively regulates the relationship between energy consumption regulation and ecological transformation, and the more funds raised through equity financing, the more significant the effect of energy consumption regulation on the improvement of resource-saving level and environment-friendly level.
H4b: Equity financing positively regulates the relationship between energy mix regulation and ecological transition, and the more funds raised through equity financing, the more significant the effect of energy mix regulation on the environment-friendly improvement.

## 3. Research design

### 3.1 Model setting and estimation method

Ecological transformation can be divided into two categories: resource-saving and environment-friendly, so the models are constructed separately for resource-saving level and environment-friendly level. In addition to capital investment, technological progress, openness to the outside world, and profitability of enterprises, smart industrial development and energy regulation are also important influencing factors for resource-saving level, and these variables are also applicable to the improvement of environment-friendly level. Energy regulation can be divided into energy mix regulation and energy consumption regulation. There is no influence relationship between energy mix and resource saving, while environmental protection inputs are mainly oriented to the governance of non-desired outputs, and there is no obvious relationship to resource conservation, so these two variables are no longer considered in the analysis of resource saving levels. The model is set as

$$\begin{array}{c} {IE}_{it}=a_{1}{KI}_{it}+a_{2}{RDI}_{it}+a_{3}{FDI}_{it}+a_{7}{ROA}_{it}+\\ (a_{4}{IL}_{it}+a_{5}{EI}_{it}+a_{6}{EI}_{it}^{2})∙D(∙)+\lambda_{t}+\delta_{i}+\varepsilon_{it} \end{array}$$ (1)

$$\begin{array}{c} {UOE}_{it}=a_{1}{KI}_{it}+a_{2}{RDI}_{it}+a_{3}{FDI}_{it}+a_{4}{ROA}_{it}+a_{5}{EGI}_{it}\\ +(a_{6}{IL}_{it}+a_{7}ER_{it}+a_{8}{ER}_{it}^{2}+a_{9}{EI}_{it}+a_{10}{EI}_{it}^{2})∙D(∙)+\lambda_{t}+\delta_{i}+\varepsilon_{it} \end{array}$$ (2)

Where *IE*_*it*_ indicates the resource saving level of the *i*-th industry in the *t*-th year, *UOE*_*it*_ indicates the environment-friendly level, *KI*_*it*_ indicates capital investment, *RDI*_*it*_ indicates the scientific and technological progress, *FDI*_*it*_ indicates the degree of openness to the outside world, *ROA*_*it*_ indicates the industry’s own profitability, *EGI*_*it*_ indicates environmental protection input, *IL*_*it*_ indicates the level of intelligent industrial development, *ER*_*it*_ indicates energy mix regulation, *EI*_*it*_ indicates the nonlinear characteristics of energy consumption regulation whose square term is used to measure *ER*_*it*_ and *EI*_*it*_. *δ*_*i*_ indicates the industry individual fixed effect item, *λ*_*t*_ indicates whether the time fixed effect item exists or whether the time fixed effect can be determined by the dummy variable, *ε*_*it*_ indicates the random disturbance item, *a*_*i*_ coefficient indicates the regression coefficient of each variable. The selection function *D*(∙) is a continuous and bounded logic function, valued between 0 and 1. D(∙) = 0 means no selection, and D(∙) = 1 means complete selection.

Equity financing can have a better moderating effect on factor inputs and thus promote industrial development. In order to measure the regulating effect of equity on the relationship between energy regulation, ecological transformation and intelligent industry, and cross-terms are introduced into Models (1) and (2) to measure the regulating effect of regulating variables. The model is as follows:

$$\begin{array}{c} {IE}_{it}=a_{1}{KI}_{it}+a_{2}{RDI}_{it}+a_{3}{FDI}_{it}+a_{4}{ROA}_{it}+a_{5}{IL}_{it}+a_{6}{EI}_{it}+a_{7}{EI}_{it}^{2}+a_{8}{EQUI}_{it}\\ +a_{9}{{EQUI}_{it}∙IL}_{it}+a_{10}{{EQUI}_{it}∙EI}_{it}+\lambda_{t}+\delta_{i}+\varepsilon_{it} \end{array}$$ (3)

$$\begin{array}{c} {UOE}_{it}=a_{1}{KI}_{it}+a_{2}{RDI}_{it}+a_{3}{FDI}_{it}+a_{4}{ROA}_{it}+a_{5}{EGI}_{it}+a_{6}{IL}_{it}+a_{7}ER_{it}+\\ a_{8}{ER}_{it}^{2}+a_{9}{EI}_{it}+a_{10}{EI}_{it}^{2}+a_{11}{EQUI}_{it}+a_{12}{EQUI}_{it}∙{IL}_{it}+a_{13}{EQUI}_{it}∙{ER}_{it}+\\ a_{14}{EQUI}_{it}∙{EI}_{it}+\lambda_{t}+\delta_{i}+\varepsilon_{it} \end{array}$$ (4)

Where *EQUI*_*it*_ indicates equity financing in year *t* for industry *i*. And the cross terms *EQUI*_*it*_∙*IL*_*it*_, *EQUI*_*it*_∙*ER*_*it*_ and *EQUI*_*it*_∙*EI*_*it*_ indicate the moderating effect of the moderating variables on the relationship between the explanatory and explained variables.

Transcendental logarithmic function is more applied to the study of substitution relationship between elements [31–34]. The cross term in the function form can reflect the interaction and change of substitution effect between input elements without making any presupposition on the interaction of substitution bombs [34]. The introduction of some cross terms in the model can study the adjustment effect of adjustment variables [20]. This model does not adopt the complete form of transcendental logarithmic model, but only introduces the cross-term of equity and intelligent industrial energy structure regulation and energy consumption regulation to observe the regulating effect of equity variables on explanatory variables, and introduces the square term to observe the nonlinear characteristics of energy regulation.

Using Eqs (1) and (2) to estimate the model, we can study the relationship between control variables, explanatory variables and resource saving level and friendly level, while the estimation results of Eqs (3) and (4) can be used to study the moderating relationship of moderating variables. Since each variable of industrial sector has an impact on resource saving level and environmentally friendly level in ecological transformation, which are not independent processes, the perturbation terms between each equation are likely to be correlated, and the model needs to be estimated using a panel seemingly uncorrelated regression method [35], which, compared to individual equations that are independently regressed one by one, can improve the validity of the regression results [36]. All the acronyms are all listed in Table 1.

**Table 1:** Acronyms.

Acronyms	Full Names
EPI	Environmental Performance Index
COP26	Conference of the Parties to the United Nations Framework Convention on Climate Change
GTFP	Green Total Factor Productivity
IE	Resource-saving Level
UOE	Environment-friendly Level
KI	Fixed Capital Investment
RDI	R&D Investment
FDI	Degree of Openning to the World
ROA	Profitability
EGI	Environmental Protection Investment
IL	Intelligent Industry
ER	Energy Structure Regulation
EI	Energy Consumption Regulation
EQUI	Equity Financing
PPP	Public—Private—Partnership

### 3.2 Variable descriptions

In this paper, the variables are divided into explained variables, explanatory variables, moderating variables, and control variables, as shown in Table 2.

**Table 2:** Variable names, variable symbols and measurement methods.

Variable Types	Variable Names	Variable Symbols	Variable Measures
Explained Variables	Resource-saving Level	IE	Resource Input Efficiency
	Environment-friendly Level	UOE	Non-desired Output Efficiency
Explanatory Variables	Intelligent Industry	IL	Average Labor Output/Base Year Average Labor Output
	Energy Structure Regulation	ER	Share of Clean Energy
	Energy Consumption Regulation	EI	Energy Consumption/Gross Industrial Output Value
Moderating Variables	Equity Financing	EQUI	Paid-in Capital/Total Assets
Control Variables	Fixed Capital Investment	KI	Fixed Assets Stock/Gross Industrial Output Value
	R&D Investment	RDI	R&D Assets Stock/Gross Industrial Output
	Environmental Protection Investment	EGI	Environmental Management Investment/Gross Industrial Output Value
	Degree of Opening to The World	FDI	Foreign Direct Investment/Gross Industrial Output Value
	Profitability	ROA	Return on Assets

(1) Explained Variables

The goal of ecological transformation is to transform to resource-saving and environment-friendly. Therefore, resource-saving and environment-friendly levels are selected as explanatory variables and quantified using resource input efficiency and unexpected output efficiency, respectively. Resource input efficiency and non-desired output efficiency are measured using a hybrid super-efficiency SBM-DEA approach [37]. The various types of resource input are taken as input indicators for the model, with the non-desired outputs and desired outputs as output indicators for the model. The input and output matrices are denoted as *X*∈*R*^*m*×*n*^ and *Y*∈*R*^*s*×*n*^, respectively, with m defined as the number of input indicators, defined as the number of output indicators, and n defined as the number of decision units. *X* = (*X*^*R*^,*X*^*NR*^), while $X^{R}\in R^{m_{1}\times n}$ indicates the radial component of the input index and $X^{NR}\in R^{m_{2}\times n}$ indicates the non-radial component, *m* = *m*_1_+*m*_2_. $Y^{bR}\in R^{s_{21}\times n}$ indicators the radial components, $Y^{bR}\in R^{s_{21}\times n}$ indicates the non-radial components, and $Y^{bNR}\in R^{s_{22}\times n}$ indicates the non-desired output, with s = *s*_21_+*s*_22_. Desired output is defined as $Y^{g}\in R^{s_{1}\times n}$. Since only gross industrial output is used as desired output, it is not possible to distinguish between radial and non-radial. The hybrid super-efficient SBM-DEA model is expressed as

$$\delta^{*}=\min\frac{m_{1}}{m}\theta +\frac{1}{m}\sum_{i=1}^{m_{2}}\theta_{i}+\frac{s_{21}}{s_{2}}\varphi +\frac{1}{s_{2}}\sum_{i=1}^{s_{22}}\varphi_{i}$$

$$s.t.\;\theta x_{i0}^{R}\geq \sum_{j=1,\neq 0}^{n}x_{ij}^{R}\lambda_{j}\;i=1,2,\cdots ,m_{1}$$

$$\theta_{i}x_{i0}^{NR}\geq \sum_{j=1,\neq 0}^{n}x_{ij}^{NR}\lambda_{j}\;i=m_{1}+1,\cdots ,m$$

$$y_{i0}^{g}\leq \sum_{j=1,\neq 0}^{n}y_{ij}^{g}\lambda_{j}\;i=1,2,\cdots ,s_{1}$$ (5)

$$\varphi y_{i0}^{bR}\geq \sum_{j=1,\neq 0}^{n}y_{ij}^{bR}\lambda_{j}\;i=s_{1}+1,\cdots ,s_{1}+s_{21}$$

$$\varphi_{i}y_{i0}^{bNR}\geq \sum_{j=1,\neq 0}^{n}y_{ij}^{bNR}\lambda_{j}\;i=s_{1}+s_{21}+1,\cdots ,s$$

$$\theta \geq 0,\theta_{i}\geq 0,\varphi \geq 0,\varphi_{i}\geq 0,\lambda_{i}\geq 0(\forall i).$$

The resource input efficiency is expressed as:

$$IE=\frac{m_{1}}{m}\theta^{*}+\frac{1}{m}\sum_{i=1}^{m_{2}}\theta_{i}^{*}$$ (6)

The undesirable output efficiency is expressed as:

$$UOE=\frac{s_{21}}{s_{2}}\varphi^{*}+\frac{1}{s_{2}}\sum_{i=1}^{s_{22}}\varphi_{i}^{*}$$ (7)

In order to make resource input efficiency and undesired output efficiency indicate that DEA is effective at the value of 1, the weight of efficiency values of each index in the model is adjusted. When the efficiency value is less than 1, it indicates non-DEA efficiency, and when the efficiency value is greater than 1, it indicates super efficiency. Therefore, the efficiency value can represent the level of resource saving and environmental friendliness in ecological transformation. Each industry can be classified according to the value of resource input efficiency and unexpected output efficiency. If both of them are greater than 1, it means low consumption and low emission and if both of them are less than 1, it means high consumption and high emission. To calculate the efficiency of resource input and undesirable output, the Malmquist index method is needed, which can be expressed as follows:

$$MI={\left[\frac{\delta^{t_{1}}((x_{0},y_{0}{)}^{t_{2}})}{\delta^{t_{1}}((x_{0},y_{0}{)}^{t_{1}})}\times \frac{\delta^{t_{2}}((x_{0},y_{0}{)}^{t_{2}})}{\delta^{t_{2}}((x_{0},y_{0}{)}^{t_{1}})}\right]}^{\frac{1}{2}},$$ (8)

where (*x*_0_, *y*_0_) refer to the evaluated DMU, *t*_1_ refers to times of evaluation groups in panel data and *t*_2_ refers times when DMU is evaluated.

In the selection of input-output indicators, energy, water, land, fixed capital and human capital are generally used as input indicators of resource input efficiency and output efficiency. These five indicators are mostly used for ecological input of regional dimension [38–40]. For industrial industries, land index is also applicable. Therefore, in this study, energy consumption, industrial water consumption, fixed capital input and human capital input are used as input indicators, and the expected output index is the total industrial output value [41,42]. The total discharge of waste water, waste gas and solid waste is generally selected as the undesirable output index, or only the main pollutant index is used.

(2) Explanatory variable, moderating variable and control variable

The explanatory variables are intelligent industry and energy regulation, in which intelligent industry uses intelligent level to quantify. Intelligence is the further upgrade of industrial automation, industrial automation is also a development stage of intelligent industry, intelligent industry is the result of information and the application of new technology, the main feature is a substantial reduction in human capital and production efficiency. Since 1993–1996 is an important stage of Chinese information construction and the beginning of industrial automation, 1996 can be regarded as the initial year of intelligent industry, intelligent development level can be expressed by using the increase times of average labor output, namely, "average labor output/average annual labors output". Energy regulation is divided into energy structure regulation and energy consumption regulation. Energy structure regulation is quantified by "proportion of clean energy consumption in total energy consumption". Energy consumption regulation is a policy constraint on total energy consumption control, which requires greatly reducing energy intensity and improving energy efficiency. Hence the use of "energy consumption per unit of GDP" to quantify.

Moderating variables select equity financing indicators to study the adjustment effect of equity financing on explanatory variables, and use "the proportion of paid-in capital of the industry in total assets of the year" to quantify.

The control variables are fixed capital investment, R&D investment, environmental protection investment, openness to the outside world, profitability and other commonly used influencing factors. Fixed capital input is expressed as "fixed assets stock/gross industrial output", and fix capital stock is expressed as "total fixed assets". R&D input is represented by "R&D assets stock/total industrial output value", the inventory of R&D assets is calculated using the perpetual inventory method, and the depreciation rate is estimated by Lin Yun [43]. The investment in environmental protection is expressed by the sum of the investment in the treatment of three wastes/the total industrial output value, that is, the investment in environmental protection required per unit of output. The degree of opening to the outside world is expressed as "foreign direct investment/gross industrial output". Profitability uses a measure of return on assets known as "net profit/total assets".

### 3.3 Sample selection and data source

Industrial industry is an important part of China’s economic development, the total industrial energy consumption accounts for more than 65% of the total energy consumption, more than 80% of sulfur dioxide and smoke (powder) dust emissions from industrial industry, nitrogen oxide emissions account for more than 60%, industrial industry brings huge resource consumption, but also produces serious pollution problems. Therefore, the industrial ecological transition is more urgent, this paper aims at the industrial sector of our country to study. With reference to the classification results of Guo et al. [44] and the data of statistical Yearbook, China’s industrial sectors are divided into 21 categories, and the classification results are shown in Table 3.

**Table 3:** Division of China’s industrial sectors.

Industry code	Industry name	Industry code	Industry name
S1	Coal mining and dressing industry	S12	non-metallic mineral products industry
S2	Petroleum and gas extracting industry	S13	Metal smelting and rolling processing industry
S3	Metals mining and processing industry	S14	Metal Products Industry
S4	Non-metallic mining and processing industry	S15	General and special equipment manufacturing industry
S5	Food manufacturing and tobacco processing industry	S16	Transportation equipment manufacturing industry
S6	Textile industry	S17	Electrical, mechanical and equipment manufacturing industry
S7	Garment leather down and its products industry	S18	Communication equipment, computer and other electronic equipment manufacturing industry
S8	Timber processing and furniture manufacturing	S19	Instrument and cultural office machinery manufacturing
S9	Paper printing and cultural and educational supplies manufacturing industry	S20	Electricity and heat production and supply industry
S10	Petroleum processing, coking and nuclear fuel processing industries	S21	Gas and water production and supply industry
S11	Chemical industry		

The data of each index are from China Statistical Yearbook, China Industrial Statistical Yearbook, China Environmental Statistical Yearbook, and China Science and Technology Statistical Yearbook. The data of some missing years are supplemented by the regression method of consumption or emission trend per unit output. The data collection scope is from 2008 to 2020, and the data related to prices are converted using 2008 constant prices. The descriptive statistics of the data are shown in Table 4.

**Table 4:** Descriptive statistics.

	IE	UOE	KI	RDI	EGI	FDI	IL	ER	EI	ROA	EQUI
Average value	0.831	0.353	0.449	0.055	0.00222	0.024	7.667	0.281	0.411	0.246	0.238
Standard deviation	0.365	0.429	0.499	0.034	0.00297	0.0202	3.433	0.118	0.364	0.135	0.202
Maximum value	1.895	1.983	4.663	0.167	0.0178	0.111	18.2	0.64	1.931	0.613	2.027
Minimum value	0.251	0.022	0.139	0.012	8.93E-05	3.20E-05	1.884	0.0575	0.0401	-0.201	0.107

## 4. Analysis of empirical results

### 4.1 Measurement of ecological transformation

**Fig 1:**
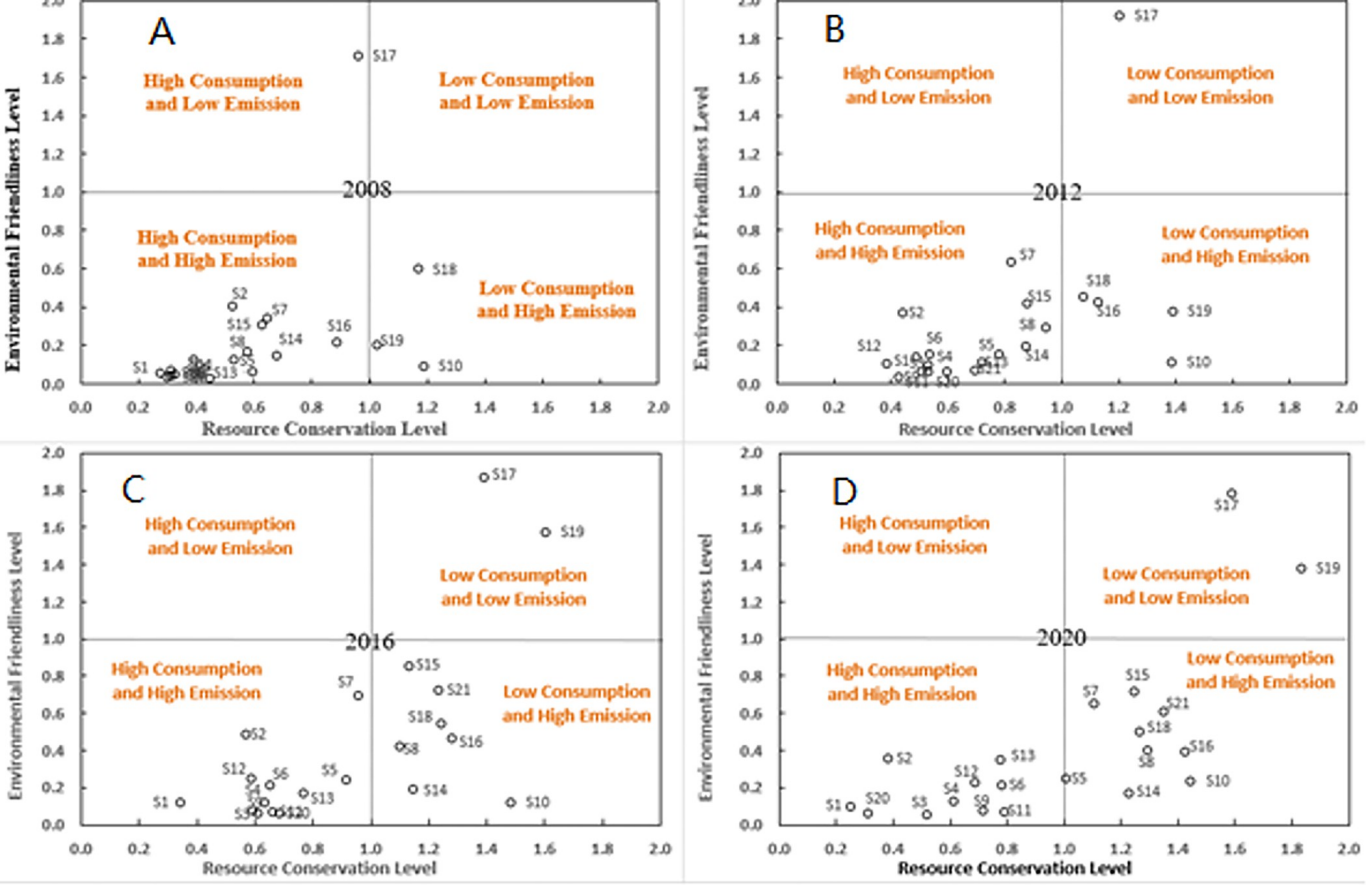
Ecological transition trend of Chinese industry. (A) Resource conservation and environmental friendliness level of 21 industrial sectors in China in 2008;(B) Resource conservation and environmental friendliness level of 21 industrial sectors in China in 2012; (C) Resource conservation and environmental friendliness level of 21 industrial sectors in China in 2016; (D) Resource conservation and environmental friendliness level of 21 industrial sectors in China in 2020.

IE and UOE can be measured according to Eqs (5)–(8), and some results are shown in Fig 1.

According to the effectiveness of DEA in resource conservation and environmental friendliness, 21 industrial sectors can be divided into four categories: low consumption and low emission, high consumption and high emission, low consumption and high emission, and high consumption and low emission. As shown in Fig 1A, in 2008, most industries in China belong to high consumption and high emission type, with only S17 belonging to high consumption and low emission type, S10, S18 and S19 belonging to low consumption and high emission type, and no industry reaching the level of low consumption and low emission. As shown in Fig 1B and 1C, With the passage of time, the trend of industrial sectors transforming to low consumption type is more obvious, and the trend of transforming to low emission type has increased, but the vast majority of industries are still in non-DEA efficient state.As shown in Fig 1D, in 2020, there are 11 industries of low consumption type, 8 more than in 2008, among which 2 industries of low consumption and low emission type (S17 and S19), and 10 industries are still of high consumption and high emission type, that is, they have not reached DEA effectiveness in terms of resource conservation and environmental friendliness. It can be seen that China’s industrial industry has an obvious transition trend to resource-saving type, while the transition to environment-friendly type is slow.

### 4.2 Test the relationship between intelligent industry, energy regulation and ecological transformation

In order to verify the hypotheses proposed in this paper, the seemingly unrelated regression is used to test the impact of smart industry, energy regulation and equity financing on ecological transformation, and the results are shown in Tables 5 and 6. Model 1 (M1) uses the relationship model with D(∙) = 0 in Eqs (1) and (2), and only tests the impact of control variables on ecological transition. Model 2 (M2) adds explanatory variables on the basis of M1, and uses the relationship model with D(∙) = 1 in Eqs (1) and (2) to test the impact of adding explanatory variables in the model. Model 3 (M3) uses Eqs (3) and (4) to test the moderating effect of equity financing on explanatory variables. The results of BP test show that the χ2 values of the three models are 78.948, 57.324 and 47.167, respectively, which are significant at the 1% level, indicating that there is significant contemporaneous correlation in the residual terms, and it is suitable to use the quasi-uncorrelated regression for estimation.

**Table 5:** Relationship test of intelligent industry, energy regulation and resource conservation level.

	Model 1 (M1)	Model 2 (M2)	Model 3 (M3)
Control variable			
KI	-0.072***(-2.873)	-0.097***(-4.351)	-0.100**(-2.354)
RDI	1.644***(3.665)	2.272***(5.866)	2.506***(5.489)
FDI	-12.351***(-18.849)	-6.021***(-8.582)	-5.354***(-5.684)
ROA	-0.093(-1.312)	0.150***(2.633)	0.167***(2.683)
Explanatory variable			
IL		0.034***(10.795)	0.031***(7.574)
EI		-0.231*(-1.685)	-0.237*(-1.706)
EI2		0.143***(2.700)	0.134**(2.256)
Moderating variable			
EQUI			-0.189(-0.752)
Interaction			
EQUI×IL			0.020*(1.799)
EQUI×EI			0.066(0.503)
R-squared	0.825	0.859	0.868
BP test	78.948***	57.324***	47.167***

***, ** and * represent significant at the significance level of 1%, 5% and 10%, respectively.

**Table 6:** Relationship test of the relationship between smart industry, energy regulation and environmental friendliness level.

	Model 1 (M1)	Model 2 (M2)	Model 3 (M3)
Control variable			
KI	-0.056(-1.628)	-0.031(-0.779)	-0.145**(-1.973)
RDI	4.358***(8.096)	5.369***(8.019)	6.839***(9.248)
FDI	-5.784***(-7.105)	-4.058***(-3.662)	-0.650(-0.442)
ROA	0.234***(2.603)	0.273**(2.548)	0.249**(2.187)
EGI	1.281(0.167)	11.109(1.262)	4.043(0.458)
Explanatory variable			
IL		0.014**(2.539)	-0.001(-0.100)
ER		-0.112(-0.217)	1.003*(1.748)
ER^2^		-0.767(-1.054)	-2.866***(-2.979)
EI		-0.235(-1.189)	-0.017(-0.080)
EI^2^		0.103(1.270)	-0.016(-0.166)
Moderating variable			
EQUI			-1.432***(-3.038)
Interaction			
EQUI×IL			0.082***(2.953)
EQUI×ER			1.449**(2.360)
EQUI×EI			0.319(1.384)
R-squared	0.462	0.478	0.510
BP test	78.948***	57.324***	47.167***

***, ** and * represent significant at the significance level of 1%, 5% and 10%, respectively.

Before adding the explanatory variables to the relationship model of resource conservation level, R^2^ was 0.825, which increased to 0.859 after adding explanatory variables and 0.868 after adding moderating variables. The explanatory power increased from 82.5% to 85.9% and 86.8%, respectively. In the environmental friendliness level relationship model, R^2^ is 0.462 before adding explanatory variables, and it increased to 0.478 after adding explanatory variables, and to 0.51 after adding moderating variables. Therefore, the explanatory power of the model is enhanced by adding explanatory variables and moderating variables.

In the control variables, the negative effect of KI to resource conservation is statistically significant at 5%, but is insufficient to environmental friendliness, indicating that the scale expansion of low-end industrial capacity from 2008 to 2020 has deteriorated the ecological environment. RDI has a significant positive impact on resource conservation and environmental friendliness. FDI has a significantly negative impact on resource conservation and environmental friendliness (Song et al. [3] found that when a certain threshold is reached, the degree of openness will reduce the growth of green GDP), indicating that the impact of FDI on resource conservation and environmental friendliness has begun to exceed the threshold and has a negative impact. ROA has a significantly positive impact on resource conservation and environmental friendliness, indicating that the improvement of profit margin promotes the behavioral decision of resource utilization efficiency and pollution emission improvement in the industrial sector. EGI is also has a positive effect on environmental friendliness, but the it is insufficient.

The explanatory variable IL has a significantly positive impact on the levels of resource conservation and environmental friendliness (both pass the significance test of 1%), indicating that the development of intelligent industry helps to improve the efficiency of resource utilization and reduce the undesirable output, thus promoting the transformation of the industrial industry to resource-saving and environment-friendly. The regression coefficient of EI2 is positive and is statistically significant at1%, indicating that there are nonlinear characteristics in energy consumption regulation, that is, there is a U-shaped relationship between energy consumption regulation and resource conservation. The coefficient of EI is negative statistically significant at 10% 10%, indicating that with the continuous strengthening of energy consumption regulation, energy intensity shows a decline, thus promoting the improvement of resource utilization technology. For the level of environmental friendliness, different from previous scholars’ research in the United States, the European Union [45] and Spain [46], the regression coefficients of EI2 and ER2 are not significant, nor for EI and ER, thus hypotheses H2b and H2c cannot be supported.

The regulatory variable equity finance has a significant regulatory effect on IL and the regression coefficients of EQUI×IL for IE and UOE are both positive, and significant at 10% and 1% respectively, which suggests that equity finance can positively regulate the relationships among intelligent industry and resource conservation and environmental friendliness. That is, the more funds raised through equity finance, the more significant the promotion effect of intelligent industry on resource conservation and environmental friendliness. Therefore, the assumption H3 is supported. The regression coefficients of EQUI×EI for IE and UOE are both positive, but are not significant. Therefore, the positive regulatory effects of equity finance on energy consumption regulation and resource conservation and environmental friendliness are not significant, and Hypothesis H4a cannot be fully verified. The regression coefficient of EQUI×ER for UOE is positive and significant at 5%, meaning that the positive regulatory effect of equity finance on energy structure regulation and the level of environmental friendliness has been tested. That is, the more equity finance, the better the energy structures regulation intensity can promote the improvement in environmental friendliness. Thus, Hypothesis H4b is verified.

The characteristics of China’s industrial industry’s transition from high consumption and high emissions to low consumption during 2008–2020 are relatively obvious, presented in Fig 1. The estimations are based on the classification results of industry types in 2020, and the results are shown in Table 7. We can see that the impact of various factors on ecological transformation in low consumption industries is obviously better than that in high consumption and high emissions industries, which is the main reason for the rapid ecological transformation of low consumption industries. In industries with high consumption and high emissions, the regression coefficients of ER2 and EI2 for UOE are negative and significant, suggesting that the impact of energy regulation on the level of environmental friendliness in high consumption and high emissions industries presents a significant non-linear characteristic. Among them, the regression coefficient of ER is positive and significant, showing that strengthening the regulation of energy structure can better improve the level of environmental friendliness, while the regression coefficient of EI is positive and significant, indicating that strengthening the regulation of energy consumption has not caused the improvement of environmental friendliness, and is in the inhibition stage. Therefore, in high consumption and high emission industries, H2b and H2c are verified. Energy regulations in low consumption industries also have similar characteristics, but they are not significant, meaning that energy consumption regulations have not significantly crossed the "threshold", so H2b and H2c are not fully supported in low consumption industries. The regulatory effect of equity finance in low consumption industries is obviously stronger than that in high consumption and high emissions industries, showing that low consumption industries can use more equity finance for the development of intelligent industries and the implementation of energy regulations, thereby better promoting ecological transformation. In low consumption industries, the regression coefficients of EQUI×EI for IE and UOE are positive and significant, so equity finance has significant positive regulatory effects on energy consumption regulation and the levels of resource conservation and environmental friendliness. Thus, Hypothesis H4a is valid in low consumption industries, but not in high consumption and high emissions industries.

**Table 7:** Estimated results for different types of industries.

	IE		UOE	
	High consumption and high emission type	Low consumption type	High consumption and high emission type	Low consumption type
KI	-0.056***(-4.182)	0.065(1.078)	0.024*(1.691)	-0.329*(-1.831)
RDI	0.828*(1.899)	2.423***(6.540)	-1.162***(-2.584)	6.959***(7.383)
FDI	-3.542***(-5.770)	0.537(0.639)	0.175(0.276)	-3.655*(-1.869)
ROA	0.139***(3.197)	0.361***(5.081)	0.093**(2.129)	0.299(1.408)
EGI			2.294(0.724)	-42.960(-1.175)
IL	0.024***(11.240)	0.065***(13.210)	0.007***(2.970)	0.048***(3.671)
ER			1.180***(4.587)	1.407(1.337)
ER^2^			-1.224***(-3.854)	-4.695***(-2.732)
EI	-0.120(-1.365)	-1.765***(-5.157)	0.135*(1.695)	0.611(1.070)
EI^2^	0.047(1.508)	0.599***(2.706)	-0.064**(-2.176)	-0.207(-0.552)
EQUI	0.467***(2.714)	-1.183***(-3.030)	0.266(0.946)	-5.749***(-4.177)
EQUI×IL	-0.038***(-3.012)	0.030(1.492)	-0.015(-1.011)	0.214***(3.690)
EQUI×ER			-0.319(-0.820)	8.466***(3.162)
EQUI×EI	-0.203**(-2.390)	0.929***(3.239)	0.009(0.101)	2.191**(2.568)

Note

***, ** and * show significant at 1%, 5%, and 10% significance levels, respectively.

## 5. Conclusion and enlightenment

In the context of the intelligent and ecological transformation of China’s industrial industry, this article introduces the variables of intelligent industry, energy regulation, and equity finance to empirically study the relationship among intelligent industry, energy regulation and ecological transformation, which is based on the data of 21 industrial industries in China from 2008 to 2020 and in the two dimensions of ecological transformations from resource conservation and environmental friendliness. The main conclusions are summarized as follows:

(1) From 2008 to 2020, China’s industrial industry has shown a trend of gradual improvement in both dimensions of resource conservation and environmental friendliness, but the ecological transformation situation is still severe, and the speed of transition to resource conservation is obviously faster than that of environmental friendliness. In 2008, there were 17 high consumption and high emission industries, and in 2020, there were 11 low consumption and high emission industries. However, there were still 10 high consumption and high emission industries, and only 2 low emission industries. Most industries still need to make strong efforts to reduce emissions to improve their environmental friendliness.
(2) The development of intelligent industry has a significant positive driving effect on ecological transformation, while equity finance has a significant positive regulatory effect on the relationship between intelligent industry and ecological transformation. It can be seen that the more equity finance, the more inclined the industrial industry is to increase investment in intelligent transformation, which can better achieve ecological transformation.
(3) There are differences in the effect of energy consumption regulation and energy structure regulation in ecological transformation. The intensity of energy consumption regulation can have a significant stimulating effect on the transformation of resource conservation, while it is still in the inhibition stage for the transformation of environmental friendliness. This inhibition feature is more significant in high consumption and high emission industries. Energy structure regulation can have a significant stimulating effect on the transformation of environmental friendliness, and no inhibitory characteristics have been observed in various industries. Equity finance has a significant positive regulatory effect on energy structure regulation and the level of environmental friendliness. The more equity finance, the stronger energy structure regulation can better promote the transformation of environmental friendliness. The regulatory effect of equity finance on energy consumption regulations is not significant enough in the industry as a whole, but it is more significant and obviously stronger in low consumption industries than in high consumption and high emissions industries, showing that equity finance can better promote the implementation of energy consumption regulations and accelerate ecological transformation.

we propose the following policy recommendations: ①Accelerate the intelligent transformation of traditional industries. Traditional industries need to promote the integration of new generation information technology and industry faster, improve the chain of new technology industry, and expand intelligent service based high value-added businesses. At the same time, it is necessary to attach importance to research and development innovation, transform from independent enterprise innovation to cross domain collaborative and networked innovation systems, and fully leverage the advantages of industrial clustering. ②Improve the design of green innovation policies. We should appropriately increase the intensity of energy structure regulation, promote the impact of energy consumption regulation on the impact breakthrough "threshold" of environmental friendliness level faster, and achieve a positive incentive effect. At the same time, we are supposed to pay attention to differences in regional economy, resource endowment and other factors, and adopt a policy design idea that combines inverted coercions and incentives. ③Make full use of the regulatory role of equity finance. On one hand, we can vigorously promote the "PPP" model of green technology innovation and intelligent upgrading, improve the enthusiasm of private enterprises and social capital to participate, and guide more equity capital into the process of intelligent and ecological transformation. On the other hand, we can formulate additional equity or subsidy support policies to increase credit support for high-risk activities such as green technology innovation and help the industrial industry improve the finance environment as well as alleviate finance pressure.

## References

[1] Huang J, Chen J. Ecological Civilization: Conceptual System and Internal Logic [J]. Journal of China University of Geosciences (Social Science Edition). 2012, 12(4): 26–30.

[2] Fan D, Sun X T. Environmental Regulation, Green Technology Innovation and Green Economic Growth [J]. China’s Population, Resources, and Environment. 2020, 30(6): 105–115.

[3] Song X, Zhou Y, Jia W. How do Economic Openness and R&D Investment Affect Green Economic Growth? -Evidence from China[J]. Resources, Conservation & Recycling. 2019, 146: 405–415.

[4] Fu F, Ma L, Li Z, et al. The implications of China’s investment-driven economy on its energy consumption and carbon emissions[J]. Energy Conversion and Management. 2014, 85: 573–580.

[5] Liu F., Sim J. Y., Sun H., et al.Assessing the role of economic globalization on energy efficiency: Evidence from a global perspective[J/OL]. China Economic Review, 2023, 77: 101897.

[6] Muganyi T, Yan L, Yin Y, et al. Fintech, regtech and financial development: evidence from China[J/OL]. Financial Innovation. 2022, 8(1): 29.

[7] Adebayo T S, Kartal M T, Ağa M, et al, Role of country risks and renewable energy consumption on environmental quality: Evidence from MINT countries[J/OL]. Journal of Environmental Management, 2023, 327: 116884. 36473361 10.1016/j.jenvman.2022.116884

[8] Akram R., Ibrahim R L, Wang Z, et al. Neutralizing the surging emissions amidst natural resource dependence, eco-innovation, and green energy in G7 countries: Insights for global environmental sustainability[J/OL]. Journal of Environmental Management, 2023, 344: 118560. 37423021 10.1016/j.jenvman.2023.118560

[9] Alola A A, Adebayo T S Analysing the waste management, industrial and agriculture greenhouse gas emissions of biomass, fossil fuel, and metallic ores utilization in Iceland[J/OL]. Science of The Total Environment, 2023, 887: 164115. 37172848 10.1016/j.scitotenv.2023.164115

[10] Adebayo T S, Ullah S., 2023. Formulating sustainable development policies for China within the framework of socioeconomic conditions and government stability[J/OL]. Environmental Pollution, 2023, 328: 121673. 37085107 10.1016/j.envpol.2023.121673

[11] Ullah S, Adebayo T S, Irfan M., et al. Environmental quality and energy transition prospects for G-7 economies: The prominence of environment-related ICT innovations, financial and human development[J/OL]. Journal of Environmental Management, 2023, 342: 118120. 37182483 10.1016/j.jenvman.2023.118120

[12] Liu X, Adebayo T S, Ramzan M., et al. Do coal efficiency, climate policy uncertainty and green energy consumption promote environmental sustainability in the United States? An application of novel wavelet tools[J/OL]. Journal of Cleaner Production, 2023, 417: 137851.

[13] Hu B., Alola A.A., Tauni M.Z., et al. Pathway to cleaner environment: How effective are renewable electricity and financial development approaches? [J/OL]. Structural Change and Economic Dynamics, 2023, 67: 277–292.

[14] Zeng Q, Zeng X F, Yue J X. Industrial Structure, Environmental Regulation, and Environmental Quality: A Theoretical and Empirical Analysis Based on the Inter-provincial Perspective in China [J]. Business Review. 2020, 32(5): 65–75.

[15] Yao Z F. Environmental Regulation, Agricultural Investment, and Convergence of Agricultural Environmental Efficiency: An Analysis of the "Porter Hypothesis" and the Integrated Framework of Investment Adjustment Costs [J]. Statistical Research. 2020(7): 1–15.

[16] Zhang J, Li H, Xia B, et al. Impact of environment regulation on the efficiency of regional construction industry: A 3-stage Data Envelopment Analysis (DEA)[J]. Journal of Cleaner Production. 2018, 200(1): 770–780.

[17] Ju K Y, Zhou D J, Wu J M. Can environmental regulation be "win-win": Research on the Strong "Porter Hypothesis" from the Perspective of Industrial Segmentation in China [J]. Journal of Beijing Institute of Technology (Social Sciences Edition). 2020(1): 21–28.

[18] D’Orazio P, Valente M. The role of finance in environmental innovation diffusion: An evolutionary modeling approach[J]. Journal of Economic Behavior and Organization. 2019, 162: 417–439.

[19] Liu J S, Zhang J. Research on the Effect of Equity Finance on Innovation Mode Selection: Based on the Intermediary Effect of Innovation Investment [J]. Scientific Management Research. 2019, 37(6): 105–112.

[20] Wang B C, Ma S, Ma Y. Environmental Regulation, Environmental Investment, and Innovation of Sustainable Business Models in Enterprises: Using Equity Investment as the Adjusting Variable [J]. Soft Science. 2020, 34(4): 44–50.

[21] Zhong R Y, Xu X, Klotz E, et al. Intelligent Manufacturing in the Context of Industry 4.0: A Review[J]. Engineering. 2017, 3: 616–630.

[22] Chen X Y, Liu S. An Empirical Study on the Impact of Intelligent Manufacturing Transformation on Industrial Structure Upgrading [J]. Statistics & Decision. 2020(13): 121–124.

[23] Wu W Y, Liu J Y. A Research on the Mechanism and Path of Intelligent Manufacturing Promoting China’s Industrial Transformation and Upgrade [J]. Journal of Xi’an University of Finance and Economics. 2020, 33(3): 19–26.

[24] Bag S, Yadav G, Wood L C, et al. Industry 4.0 and the circular economy: Resource melioration in logistics[J]. Resources Policy. 2020, 68.

[25] Gao G. Interaction between Intelligent Manufacturing and Energy Transformation in the New Industrial Revolution [J]. Scientific Management Research. 2017, 35(5): 45–48.

[26] Chi R Y, Mei X M, Ruan H P. How does intelligent manufacturing match organizational change in small and medium-sized enterprises? [J]. Studies in Science of Science. 2020, 38(7): 1245–1250.

[27] Qian J. A Research on the threshold effect of energy conservation biased technological progress on industrial energy conservation and emission reduction [J]. Science Research Management. 2020, 41(1): 223–233.

[28] Wen Y, Ma Z, Wu Y H, et al. Decomposition of Industrial Air Pollution Emission Factors in Beijing, Tianjin, Hebei, and Surrounding Areas: Based on LMDI Model Analysis [J]. China Environmental Science. 2018, 38(12): 4730–4736.

[29] Deng X L, Che M H, Chen B D. Analysis of Environmental Pollution Effects and Influencing Factors of Urbanization in China [J]. Inquiry into Economic Issues. 2017(1): 31–37.

[30] Feng Z, Wang M, Huang H. Equity Finance and Social Responsibility: Further International Evidence[J]. The International Journal of Accounting. 2015, 50(3): 247–280.

[31] Pablo-Romero M D P, Gómez-Calero M D L P. A translog production function for the Spanish provinces: Impact of the human and physical capital in economic growth[J]. Economic Modelling. 2013, 32: 77–87.

[32] Lin B, Atsagli P. Inter-fuel substitution possibilities in South Africa: A translog production function approach[J]. Energy. 2017, 121: 822–831.

[33] Tong J P, Qin T, Ma J F, et al. Jiangsu Energy Rebound Effect Based on Random Frontier Transcendental Logarithmic Function [J]. Systems Engineering. 2015, 33(1): 139–145.

[34] Wang B B, Qi S Z. Biased Technological Progress, Factor Substitution, and Industrial Energy Intensity in China [J]. Economic Research Journal. 2014(2): 115–127.

[35] Zellner A. An Efficient Method of Estimating Seemingly Unrelated Regression Equations and Tests for Aggregation Bias[J]. Journal of the American Statistical Association. 1962, 57: 346–369. doi: 10.2307/2281644

[36] Fan M Q, Ren R E, Chen G C. An Empirical Study on the Impact of Technological Change, Factor Substitution, and Trade on Energy Intensity [J]. China Economic Quarterly. 2010(1): 237–258.

[37] Wang X, Ding H, Liu L. Eco-efficiency measurement of industrial sectors in China: A hybrid super-efficiency DEA analysis[J]. Journal of Cleaner Production. 2019, 229: 53–64. doi: 10.1016/j.jclepro.2019.05.014

[38] Huang J, Xia J, Yu Y, et al. Composite eco-efficiency indicators for China based on data envelopment analysis[J]. Ecological Indicators. 2018, 85: 674–697.

[39] Ren S, Li X, Yuan B, et al. The effects of three types of environmental regulation on eco-efficiency: a cross-region analysis in China[J]. Journal of Cleaner Production. 2018, 173: 245–255.

[40] Zhou C, Shi C, Wang S, et al. Estimation of eco-efficiency and its influencing factors in Guangdong Estimation of eco-efficiency and its influencing factors in Guangdong province based on Super-SBM and panel regression models[J]. Ecological Indicators. 2019, 86: 67–80.

[41] Yu S, Liu J, Li L. Evaluating provincial eco-efficiency in China: an improved network data envelopment analysis model with undesirable output[J]. Environmental Science and Pollution Research. 2019, 27: 6886–6903. 31879879 10.1007/s11356-019-06958-2

[42] Song M, Peng J, Wang J. Better resource management: An improved resource and environmental efficiency evaluation approach that considers undesirable outputs[J]. Resources, Conservation and Recycling. 2018, 128: 197–205.

[43] Lin Y. Accounting for R&D Capital Stock in China’s Industrial Industries [D]. Nanjing University of Finance & Economics, 2015.

[44] Guo Y, Zeng Z, Tian J, et al. Uncovering the strategies of green development in a Chinese province driven by reallocating the emission caps of multiple pollutants among industries[J]. Science of The Total Environment. 2017, 607–608: 1487–1496. 10.1016/j.scitotenv.2017.06.234 28787800

[45] Özkan O., Alola A.A., Adebayo T.S. Environmental benefits of nonrenewable energy efficiency and renewable energy intensity in the USA and EU: Examining the role of clean technologies[J/OL]. Sustainable Energy Technologies and Assessments, 2023, 58: 103315.

[46] Adebayo T.S., 2022. Environmental consequences of fossil fuel in Spain amidst renewable energy consumption: a new insight from the wavelet-based Granger causality approach[J/OL]. International Journal of Sustainable Development & World Ecology, 2022, 29(7): 579–592.

